# Characterization of *Penicillium halotolerans* with Antagonistic Activity Against *Fusarium* Root Rot in *Astragalus membranaceus*

**DOI:** 10.3390/jof12040283

**Published:** 2026-04-17

**Authors:** Yuze Yang, Haiping Jiang, Xunjue Yang, Ke Hao, Yujia Zhao, Qingzhi Yao, Min Li

**Affiliations:** 1 College of Life Science and Technology, Inner Mongolia Normal University, Hohhot 010022, China; 15144643931@163.com (Y.Y.);; 2 Key Laboratory of Biodiversity Conservation and Sustainable Utilization in Mongolian Plateau for College and University of Inner Mongolia Autonomous Region, Hohhot 010022, China; 3 College of Grassland Science, Inner Mongolia Agricultural University, Hohhot 010011, China

**Keywords:** *Penicillium halotolerans*, root rot, antagonistic activity, metabolomics, *Astragalus membranaceus*

## Abstract

*Astragalus membranaceus* is an important perennial medicinal plant whose roots constitute its primary medicinal organ; however, its cultivation is severely constrained by root rot caused by *Fusarium oxysporum*. This study aimed to characterize differences in the rhizosphere microbiome between healthy and diseased plants, identify antagonistic microorganisms from healthy rhizosphere soils, and investigate their suppressive effects on *F. oxysporum* and the associated host metabolic responses. High-throughput sequencing was used to compare bacterial and fungal communities in the rhizospheres of healthy and diseased plants. Microorganisms were isolated from healthy rhizosphere soils and screened for antagonistic activity against *F. oxysporum*, followed by validation in pot experiments. Metabolomic analysis was further conducted to assess host metabolic responses to microbial treatment. Root rot disease significantly altered the dominant composition of rhizosphere microbial communities and was associated with reduced fungal diversity and lower bacterial richness in diseased soils. Co-occurrence network analysis revealed increased complexity in bacterial networks and strengthened positive correlations among fungal taxa under diseased conditions. A total of 81 microbial strains were isolated from healthy rhizosphere soils, among which *Penicillium halotolerans* exhibited the strongest inhibitory activity against the mycelial growth of *F. oxysporum*. Pot experiments further supported its suppressive effect on Astragalus root rot. Metabolomic analysis indicated that *P. halotolerans* treatment was associated with changes in host metabolic profiles related to energy metabolism, defense-associated protein synthesis, and nutrient uptake. Overall, this study identified *P. halotolerans* as a fungal strain with antagonistic activity against *F. oxysporum* and provided initial evidence for its association with the suppression of Astragalus root rot. These findings offer candidate microbial resources and mechanistic insights for understanding rhizosphere-associated disease suppression in *Astragalus membranaceus*.

## 1. Introduction

*Astragalus membranaceus* is a perennial herbaceous plant of the genus *Astragalus* in Leguminosae. It is a traditional medicinal plant that uses roots as medicine and is mainly cultivated in northern China, Japan, and other Asian countries [1]. *A. membranaceus* has the effects of enhancing immunity, lowering blood pressure and blood sugar, protecting the cardiovascular system, anti-oxidation and anti-tumor activities, etc., and is widely popular in Asian countries [2].

With the increasing market demand, the cultivation scale of *A. membranaceus* continues to expand. Due to the frequent application of chemical pesticides and fertilizers, lack of crop rotation systems or excessively short rotation cycles, the soil experiences an accumulation of allelopathic autotoxic substances, a decrease in enzyme activity, disruption of microbial community structure, accumulation of pathogens, and weakened disease suppression function of soil microbiota, which eventually lead to the occurrence and continuous aggravation of disease [3,4,5]. Among various diseases, root rot stands out as the most prominent one. It is primarily a soil-borne disease caused by *Fusarium* [6]. In the early stages of infection in *A. membranaceus*, the roots darken, soften, and exhibit signs of decay. In severe cases, this leads to yellowing and wilting of the leaves, ultimately resulting in the death of the plant [7]. Various methods have been attempted to control the occurrence of root rot, including the use of chemical pesticides [8,9], the breeding of disease-resistant varieties, and field management practices [10,11,12]. However, the long-term use of chemical fungicides may lead to soil contamination, disrupting the soil microbial balance and reducing fertility. Additionally, pathogens may develop drug resistance, weakening control effectiveness [13,14]. Furthermore, chemical residues may also pose risks to human health and the environment, particularly affecting the safety of medicinal materials [15,16]. Microbial-based disease suppression, as an environmentally friendly and sustainable pest management strategy, has attracted increasing attention in agricultural production, medicinal plant cultivation, and ecological protection [17,18].

Multiple studies have shown that various antagonistic microorganisms can effectively inhibit the growth of pathogenic fungi through different mechanisms and regulate the structure of soil microbial communities, thereby reducing the harm of root rot. For instance, inoculating *Trichoderma harzianum* and *Bacillus amyloliquefaciens* onto the roots of *A. membranaceus* can significantly increase the relative abundance of beneficial microbial groups in the soil, such as *Holtermanniella* and *Metarhizium*, while significantly reducing the relative abundance of pathogenic fungi such as *Fusarium* [19]. By co-inoculating the biocontrol strains *Saccharomyces cerevisiae* and zinc oxide with *A. membranaceus*, the growth of the pathogenic fungus *F. oxysporum* was significantly inhibited, thereby delaying the occurrence of root rot disease [20]. Meanwhile, in field experiments, four bacterial strains belonging to the genera *Stenotrophomonas*, *Rhizobium*, *Advenella*, and *Ochrobactrum* were found to effectively inhibit the growth of *F. oxysporum*, thereby reducing the incidence and mortality of root rot disease in *A. membranaceus* [21]. Additionally, it has been reported that *Paraburkholderia* can inhibit the growth of various plant pathogens, such as *Fusarium*, by producing antibiotics like pyrrolnitrin, 2,4-diacetylphloroglucinol (DAPG), and pyoluteorin [22]. The occurrence of root rot can also significantly alter the structure of soil microbial communities. For example, after root rot occurs in *Zanthoxylum armatum*, the number of pathogenic *Fusarium* spp. in the rhizosphere soil increases significantly, while the rhizosphere soil of healthy plants maintains a high level of beneficial microorganisms [23]. Therefore, screening for highly effective antagonistic microorganisms remains a crucial and urgent task in the current research on root rot suppression, and it has important practical significance for constructing a stable and sustainable disease prevention and control system.

Metabolomics, as an emerging omics technology, is utilized to investigate plant responses to environmental stresses, identify and quantify differential metabolites within organisms, serving as a bridge connecting genes, proteins, and phenotypes [24,25,26,27]. Currently, metabolomics has been increasingly applied in various fields of agriculture [28,29,30]. For instance, Zhu et al. revealed the defense response mechanism of soybeans to root rot by analyzing metabolic changes in soybeans infected with *Phytophthora sojae* [31]. Similarly, Ye et al. utilized metabolomics to uncover key microbial communities and metabolites associated with disease resistance in *Paris polyphylla*, providing new insights into plant defense mechanisms [32]. Therefore, metabolomics research aids in gaining a deeper understanding at the molecular level of how plants adjust their biochemical pathways to cope with external stresses, as well as their metabolic responses and adaptation mechanisms under different environmental conditions.

Therefore, this study employed high-throughput sequencing technology to compare the differences in microbial composition between the rhizosphere of healthy and diseased *A. membranaceus* plants. Using the dilution and spread plate method, antagonistic fungi against the root rot pathogen *F. oxysporum* were isolated from the rhizosphere soil. Through plate confrontation experiments, the strain with the greatest antagonistic potential was screened. Metabolomics technology was utilized to analyze the differential metabolic pathways and metabolites in *A. membranaceus* treated with the pathogen alone and with both the pathogen and *Penicillium halotolerans*. The aim of this study was to uncover the disease-suppressive mechanism associated with *P. halotolerans*, providing a theoretical basis for understanding its potential role in disease management.

## 2. Materials and Methods

### 2.1. Description of the Study Area and Materials

The research area is located in Guyang County, Baotou City, Inner Mongolia Autonomous Region, China (109°38′3″ E–110°44′42″ E, 40°42′58″ N–41°28′52″ N), with typical characteristics of a temperate continental climate. The annual average temperature is 7.2 °C, with a total annual precipitation of approximately 300 mm, and the annual sunshine duration is about 2882 h. The altitude ranges from about 1300 m to 1700 m.

### 2.2. Rhizosphere Soil Sample Collection

Three replicated plots were established within *A. membranaceus* planting area, each with a size of 100 × 100 m. Within each plot, sampling points were randomly distributed in an S-shape pattern. When sampling, the roots of *A. membranaceus* were dug out downwards until the entire root system was extracted, and the rhizosphere soil was collected. The collected soil samples were placed in sterile bags and promptly transported back in a portable refrigerator. The soil samples from each plot were mixed evenly and sieved using a 2 mm sieve. The sieved soil samples were then divided into two portions: one for high-throughput sequencing and the other for the determination of soil physicochemical properties (partially air dried and partially frozen).

### 2.3. DNA Extraction, PCR Amplification, and Illumina MiSeq Sequencing

Microbial metagenomic DNA was extracted from rhizosphere soil samples using the PowerSoil^®^ DNA Isolation Kit, and the integrity of the DNA was assessed by 1.8% (*w*/*v*) agarose gel electrophoresis. The quality of the DNA extracts was assessed using a Synergy HTX microplate reader. ITS1 (5′-TAGAAGAGAAAAGTCGTAA-3′) and ITS4 (5′-TCCTC-CGCTTWTTGWTGC-3′) were used as fungal-specific primers. For bacterial community amplification, primers 341F (5′-CCTACGGGNGGCWGCAG-3′) and 806R (5′-GACTACHVGGGTATCTAATCC-3′) were used to target the 16S rRNA gene V3–V4 region. The PCR reaction system consists of 2 × Taq Plus PCR Master Mix 10 μL, template DNA 3 μL, forward and reverse primers 0.5 μL each, and water added to 20 μL. The amplification conditions are as follows: initial denaturation at 98 °C for 4 min, followed by 30 cycles of denaturation at 94 °C for 30 s, annealing at 50 °C for 30 s, and extension at 72 °C for 100 s. After the 30 cycles, a final extension step is performed at 72 °C for 10 min. The amplified product is then stored at 4 °C. After PCR completion, the amplified products were subjected to 1.8% agarose gel electrophoresis, and the target fragments were excised and recovered using the Qiagen Gel Extraction Kit (Hilden, Germany). The library was constructed using the TruSeqNano DNA LT Library Prep Kit from Illumina (San Diego, CA, USA). The quality of the library was assessed using the Agilent High Sensitivity DNA Kit and the Quant-iT PicoGreen dsDNA Assay Kit. Paired-end sequencing was then performed on the Illumina MiSeq v3 platform (2 × 300 bp).

### 2.4. Isolation and Identification of Rhizosphere Soil Microorganisms

After gradient dilution with sterile water, soil samples were plated onto PDA medium, beef extract-peptone medium, and Gause’s No. 1 medium for the isolation of fungi, bacteria, and actinomycetes, respectively. Once pure strains were obtained, their DNA was extracted using the resin method, followed by PCR amplification. The amplified PCR products were then analyzed by 0.5% agarose gel electrophoresis. After purification, the products were subjected to bidirectional sequencing by Beijing TsingKe Biotechnology Co., Ltd. (Beijing, China).

### 2.5. Assessment of Antagonistic Activity Against F. oxysporum

Using the pathogenic fungus *F. oxysporum* BN2-2, previously screened by our research group and isolated from *A. membranaceus* plants showing root rot symptoms and preserved at the Min Li Laboratory, College of Life Science and Technology, Inner Mongolia Normal University [33], as the indicator organism, a pathogen mycelial plug was inoculated into the center of a PDA medium using an 8 mm diameter cork borer. With the mycelial plug as the center, a straight line was drawn passing through the center of the plug. Two symmetrical points, each 2.5 cm away from the center along this line, were selected for inoculating the test fungal mycelial plugs. A plate inoculated with only the pathogenic fungus served as the blank control. For bacteria and actinomycetes, inoculation was done by streaking a 2 cm line at the two symmetrical points according to previously described methods [34], while for fungi, an 8 mm mycelial plug was used for inoculation using a plate confrontation assay as described by Skidmore and Dickinson with slight modifications [35]. All plates were placed in a 28 °C constant temperature incubator for inverted cultivation. After 6 days, the colony diameters of F. oxysporum were measured. The strain with the best antagonistic effect was screened by calculating the antifungal rate, and the isolate showing the highest inhibition rate in vitro was selected for subsequent identification and pot experiments. The formula is:

(1) Inhibition Rate(%)=[(D−d)/d]×100%

D—Diameter of the inhibition zone of the test strain;

d—Diameter of the inhibition zone of the control strain.

### 2.6. Inoculation of A. membranaceus Seedlings with Penicillium halotolerans

Disinfect the seeds of *A. membranaceus* with 0.1% HgCl_2_ for 3 min, followed by 75% ethanol for 1 min. After rinsing the seeds with sterile water 3 times, plant them in plastic pots filled with a mixture of vermiculite and sandy soil at a ratio of 1:1, with 5 seeds per pot. Once the seedlings develop 2 true leaves, select those with similar growth and transplant them into new pots, with 2 seedlings per pot. Based on the in vitro antagonistic assay described above, the isolate showing the highest inhibition rate against *F. oxysporum* was selected for the subsequent pot experiment. This isolate was identified as *P. halotolerans*. Six treatment groups were established: a blank control group (CK), a group inoculated solely with *F. oxysporum* (F), a group inoculated solely with *P. halotolerans* (P), and three different concentrations (1.0 × 10^5^ spores/mL, 5.0 × 10^5^ spores/mL, and 1.0 × 10^6^ spores/mL) of *P. halotolerans* co-treated with pathogenic fungi (P1F, P2F, P3F). To avoid stress effects on the plants, 25 mL of *P. halotolerans* spore suspension was added to each pot after transplanting the seedlings. After one week of adaptation by *P. halotolerans*, an additional 25 mL of *P. halotolerans* spore suspension was added to each pot. Fifteen days after transplantation, 50 mL of *F. oxysporum* spore suspension was added to each pot to infect the seedling roots with root injury perfusion method. Each treatment consisted of 10 pots, with a total of 120 seedlings. Samples were collected 45 days later to measure seedling height and root length. Then the treated seedlings were frozen in liquid nitrogen and stored at −80 °C for metabolomics analysis.

### 2.7. Metabolomics Analysis

After collecting the seedlings, randomly mix the roots of 4 seedlings from each treatment group. Take 50 mg of the mixed root samples and add an 80% methanol solution along with 3 medium steel balls for tissue fragmentation. Subsequently, add 700 μL of 80% methanol and subject the mixture to vortex mixing. The mixture is then treated with ice-bath ultrasound for 20 min and left to stand at low temperature for 2 h. Then centrifuge the mixture at 16,000× *g* at 4 °C for 20 min, collect the supernatant, and evaporate the methanol to dryness in a high-speed vacuum concentrator. Before mass spectrometry analysis, dissolve the sample in 100 μL of 50% methanol and centrifuge under the same conditions for 15 min to obtain the supernatant.

The samples were separated using a SHIMADZU-LC30 ultra-high performance liquid chromatography system (UHPLC) (Kyoto, Japan) equipped with an ACQUITY UPLC^®^ HSS T3 chromatographic column (2.1 × 100 mm, 1.8 μm, Waters, Milford, MA, USA). The system settings included: column temperature of 40 °C, flow rate of 0.3 mL/min, and injection volume of 4 μL. The separated samples were analyzed using Thermo Scientific’s QE Plus mass spectrometer (Waltham, MA, USA), with electrospray ionization (ESI) performed using a HESI source. The instrument was operated over an *m/z* range of 70–1050 Da. Full MS scans were acquired at a resolution of 70,000 at *m/z* 200, and MS/MS scans were acquired at a resolution of 17,500 at *m/z* 200. The maximum injection time was set to 100 ms for MS and 50 ms for MS/MS. The isolation window for MS2 was 2 *m/z*, and the stepped normalized collision energy was set to 20, 30, and 40. The raw data was processed using MS-DIAL 4.9.221218 software, which included peak alignment, retention time correction, and peak area extraction. Metabolite structures were identified by matching precise mass (mass deviation < 10 ppm) and secondary spectra (mass deviation < 0.01 Da), using databases such as HMDB, MassBank, GNPS, and a self-built metabolite standard library. In the extracted data, only variables having more than 50% of nonzero measurement values in at least one group were retained. The total peak area of positive and negative ion data was normalized separately, and the data was preprocessed using the Unit Variation Scaling (UV) method for subsequent analysis.

### 2.8. Bioinformatics Analysis

Raw sequencing reads were obtained in FASTQ format and quality-filtered using Trimmomatic (version 0.33). Primer sequences were identified and removed using Cutadapt (version 1.9.1) with a maximum mismatch rate of 20% and a minimum coverage of 80%. Paired-end reads were merged using USEARCH (version 10.0) with a minimum overlap length of 10 bp, a minimum overlap similarity of 90%, and a maximum mismatch of 5 bp, and chimeric sequences were removed using UCHIME (version 8.1). For fungal ITS data, features were generated using DADA2 implemented in QIIME2 (version 2020.6), and taxonomic assignment was performed using a Bayesian method against the UNITE database (Release 8.0). Features with abundance lower than 2 were removed before downstream analysis. For bacterial 16S rRNA data, taxonomic assignment was performed against the Greengenes database (version 13.5). Alpha-diversity indices of bacterial and fungal communities, including the Chao1, Shannon, and ACE indices, were calculated in R using vegan and picante packages. Community dissimilarity among samples was assessed using the Jaccard distance and visualized with the ggplot2 package. Chord diagrams and volcano plots were generated using the circlize and EnhancedVolcano packages, respectively. Mantel tests were performed using the ggcor package. Topological features of network nodes, including degree and centrality, were calculated using the igraph package. Strongly correlated edges were retained based on correlation coefficients for network construction. Network modules were identified and characterized using the WGCNA package, with a threshold of 0.9 applied to retain strongly associated features. Key network nodes were screened according to within-module connectivity (Zi) and among-module connectivity (Pi), and the networks were visualized using Gephi software (version 0.10.1).

### 2.9. Statistical Analysis

Statistical analyses were performed using R software (version 4.4.1) and other software packages as described above. Data from the pot experiment are presented as mean ± SD. Differences in root length and plant height among treatments were analyzed using one-way analysis of variance (ANOVA) followed by Tukey’s multiple comparison test. Differences in alpha-diversity indices between healthy and diseased soils were analyzed using Student’s *t*-test. Differences in microbial community structure were evaluated by PERMANOVA with 999 permutations. For metabolomics, data were mean-centered using Pareto scaling. Multivariate statistical analyses were performed using PCA, PLS-DA, and OPLS-DA, and model overfitting was evaluated by permutation tests (n = 200). Differential metabolites were identified based on VIP > 1.0 and *p* < 0.05. When the OPLS-DA model showed poor reliability, differential metabolites were screened using Fold Change ≥ 1.5 or ≤1/1.5 together with *p* < 0.05. KEGG enrichment analysis was performed using Fisher’s exact test. In the WGCNA, module–trait correlations were calculated using Pearson correlation analysis. The value of *p* < 0.05 was considered statistically significant.

## 3. Results

### 3.1. Root Rot Disease Significantly Alters the Community Composition and Abundance Distribution of Dominant Microorganisms in the Rhizosphere Soil of A. membranaceus

**Figure 1:**
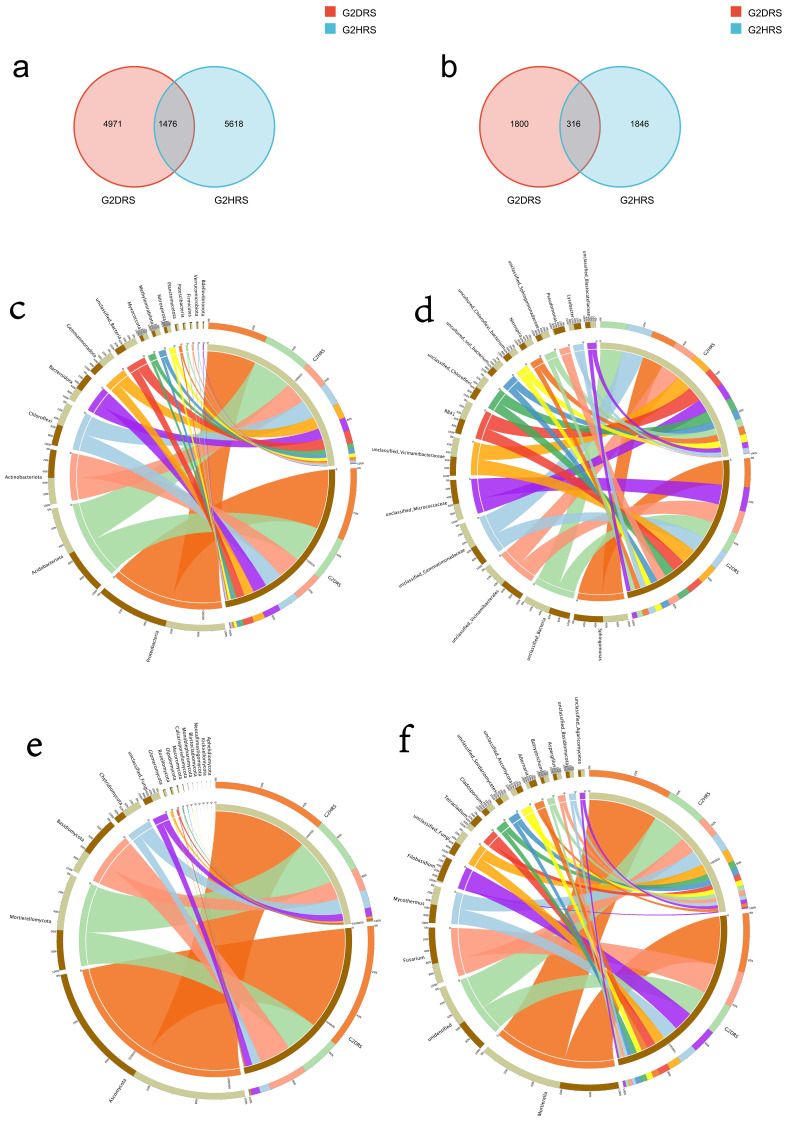
Classification and distribution of rhizosphere microorganisms in healthy/diseased *A. membranaceus*. (**a**) Bacterial Venn diagram; (**b**) Venn diagram of fungi; (**c**,**d**) Distribution map of the composition of bacteria and fungi in the rhizosphere of healthy/diseased *A. membranaceus* at the phylum level; (**e**,**f**) The composition and distribution of bacteria and fungi in the rhizosphere of healthy/diseased *A. membranaceus* at the genus level.

A total of 3962 fungal OTUs and 12,065 bacterial OTUs were obtained from the rhizosphere soil of healthy and diseased *A. membranaceus* (Figure 1a,b), among which the unique fungal and bacterial OTUs were 1800/1846 and 4971/5618, respectively. Defining phyla with a relative abundance greater than 10.00% as dominant phyla, it was observed that Proteobacteria (29.80%), Acidobacteriota (20.94%), and Chloroflexi (10.00%) are the dominant bacterial phyla in the rhizosphere soil of healthy and diseased *A. membranaceus* (Figure 1c). Similarly, Ascomycota (55.72%), Mortierellomycota (17.67%), and Basidiomycota (17.09%) are the dominant fungal phyla (Figure 1d). The genera with a relative abundance greater than 1.00% are defined as dominant genera. It can be seen that *Sphingomonas* (5.98%), *RB41* (3.71%), *Nitrospira* (1.57%), *Lysobacter* (1.33%), *Subgroup_10* (1.18%), and *P3OB-42* (1.06%) are the dominant bacterial genera (Figure 1e), while *Mortierella* (21.24%), *Fusarium* (8.38%), *Mycothermus* (5.50%), *Filobasidium* (3.85%), *Tetracladium* (2.53%), *Cladosporium* (1.95%), *Alternaria* (1.63%), *Botryotrichum* (1.23%), and *Aspergillus* (1.23%) are the dominant fungal genera (Figure 1f). These results indicate that both the fungal and bacterial communities of healthy and diseased *A. membranaceus* are consistent in the composition of dominant phyla and genera.

However, the occurrence of root rot disease significantly alters the relative abundance of dominant microorganisms. In diseased soil, the relative abundances of Glomeromycota and Myxococota significantly decreased (*p* < 0.05), while the relative abundances of Basidiomycota significantly increased (*p* < 0.05). Additionally, the relative abundances of *Mortierella*, *P3OB-42*, and *Parablastomonas* in healthy soil were significantly higher than those in diseased soil (*p* < 0.05). Conversely, the relative abundances of *Fusarium*, *Pedobacter*, *Filobasidium*, and *Neonectria* in diseased soil were significantly higher than those in healthy soil (*p* < 0.05) (Figure 1c–f).

### 3.2. Root Rot Disease Was Associated with Changes in Soil Microbial Networks and Community Interaction Patterns

**Figure 2:**
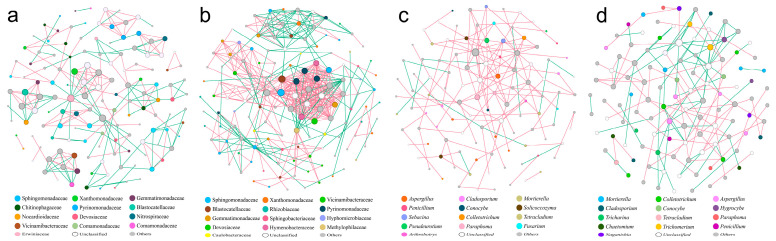
Co-occurring network of bacterial and fungal microbial communities in healthy/diseased *A. membranaceus*. (**a**) Bacterial community family level in healthy soil; (**b**) Bacterial community family level in diseased soil; (**c**) Fungal community genus level in healthy soil; (**d**) Fungal community genus level in diseased soil.

The co-occurrence network analysis of fungal and bacterial communities in healthy/diseased soils (Figure 2a–d) revealed that the occurrence of root rot significantly increases the total number of edges and average degree of each node in the bacterial network (Supplementary Table S1). After the illness, the total number of edges in the bacterial network increased by 103%, and the average degree increased by 109% (Figure 2b). In addition, the damage caused by root rot is not only reflected in the number of edges, but also in the types of microbial interactions. In the fungal network of diseased soil, positive interactions account for 48.99% of the total interactions, while in healthy soil, they only account for 9.42% (Figure 2d). Meanwhile, the occurrence of root rot disease also affects the modularity index. The modularity index of the bacterial network in diseased soil significantly decreased, while the modularity index of the fungal network in diseased soil significantly increased. The above results indicate that root rot disease alters the interactions between soil fungi and bacterial networks.

By computing the Zi and Pi values, it has been ascertained that *Fusarium* serves as a pivotal node within the diseased network (Supplementary Figure S1). A subsequent sub-network analysis focusing on *Fusarium* disclosed that, in the context of the diseased network, *Fusarium* exhibits a markedly negative correlation with a multiplicity of other fungi, namely *Typhula*, *Aspergillus*, *Conocybe*, *Clavulinopsis*, and *Colletotrichum* (Supplementary Table S2). Conversely, within the healthy network, *Fusarium* has a significantly negative correlation only with *Exophiala*, indicating a competitive or inhibitory relationship between them.

### 3.3. Root Rot Disease Weakens Soil Microbial Diversity and Reshapes Community Structure

**Figure 3:**
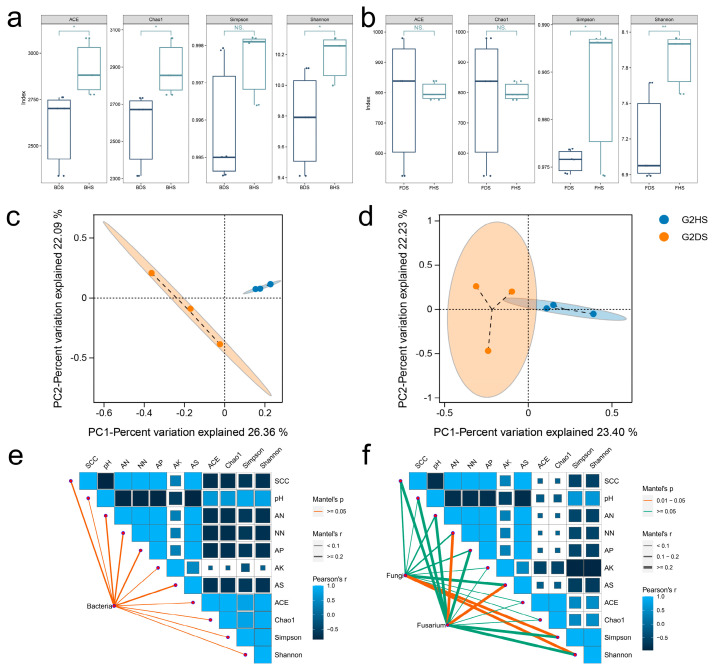
Mantel test and analysis of alpha and beta diversity of core microbial communities. (**a**) Comparison of alpha diversity of fungal core microbial communities in healthy and diseased soils; (**b**) Comparison of alpha diversity in bacterial core microbial communities; (**c**) Principal coordinate analysis based on Jaccard distance (PCoA) to demonstrate the differences in beta diversity of fungal communities; (**d**) Differences in Beta diversity of bacterial communities; (**e**) Mantel test was used to evaluate the correlation between the core microbial community of *A. membranaceus* root zone bacteria and environmental factors in healthy and diseased two-year-old individuals in Guyang; (**f**) The correlation between fungal core microbial communities and environmental factors. Note: * indicates *p* < 0.05, ** indicates *p* < 0.01.

At the lowest sequencing depth level of 90%, random resampling was performed on all samples in the OTU abundance matrix. The Simpson and Shannon indices were used to assess the diversity of microbial communities, and the ACE and Chao1 indices were used to evaluate the richness of microbial communities. The study found that both the fungal diversity and bacterial richness in diseased soil were significantly lower than those in healthy soil (Figure 3a,b). Additionally, the Principal Coordinates Analysis (PCoA) showed that the samples within the healthy/diseased soil microbial groups were relatively clustered, while the samples between the groups were relatively dispersed, indicating that the occurrence of root rot would change the composition of the soil microbial community (Figure 3c,d).

The Mantel test was conducted on the fungal community, bacterial community, and the main pathogen, *Fusarium*, in relation to environmental factors (Figure 3e,f). The results showed that *Fusarium* had a strong correlation with NN and AS. Available potassium (AK) had a significant negative correlation with fungal diversity, and the pH value had a significant positive correlation with bacterial diversity. Moreover, the pH value was negatively correlated with the vast majority of soil factors except AK.

### 3.4. Screening and Identification of Antagonistic Fungi Against the Pathogen F. oxysporum Causing Root Rot Disease

**Figure 4:**
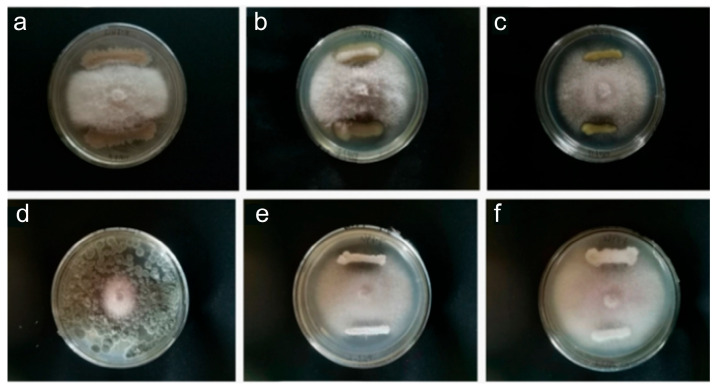
The antagonistic effects of 6 strains. (**a**) SJN3-4; (**b**)SJG3-5; (**c**) SJG3-13; (**d**) SJP3-3; (**e**) SJP3-5; (**f**) SJP3-6.

To obtain antagonistic bacteria or fungi against the pathogen *F. oxysporum*, culturable microorganisms were isolated from the roots and rhizosphere soil of *A. membranaceus*, and a total of 81 strains of bacteria and fungi were obtained. After molecular identification and removal of duplicate strains, 39 species were finally identified, including 26 species of bacteria, 5 species of actinomycetes, and 9 species of fungi (Supplementary Table S3). Through plate confrontation experiments, six strains with relatively good antagonistic effects against *F. oxysporum* were screened, namely SJN3-4, SJG3-5, SJG3-13, SJP3-3, SJP3-5, and SJP3-6 (Figure 4). The inhibition rates of these 6 strains against *F. oxysporum* were 46.49%, 35.63%, 33.33%, 57.78%, 31.25%, and 44.14%, respectively (Supplementary Table S4). Among them, the strain SJP3-3 exhibited the best antagonistic effect (Figure 4d). Based on the morphological results and molecular sequence analyses, the strain SJP3-3 was identified as *P. halotolerans*.

### 3.5. P. halotolerans Treatment Partially Alleviated Pathogen-Induced Growth Suppression in A. membranaceus

**Figure 5:**
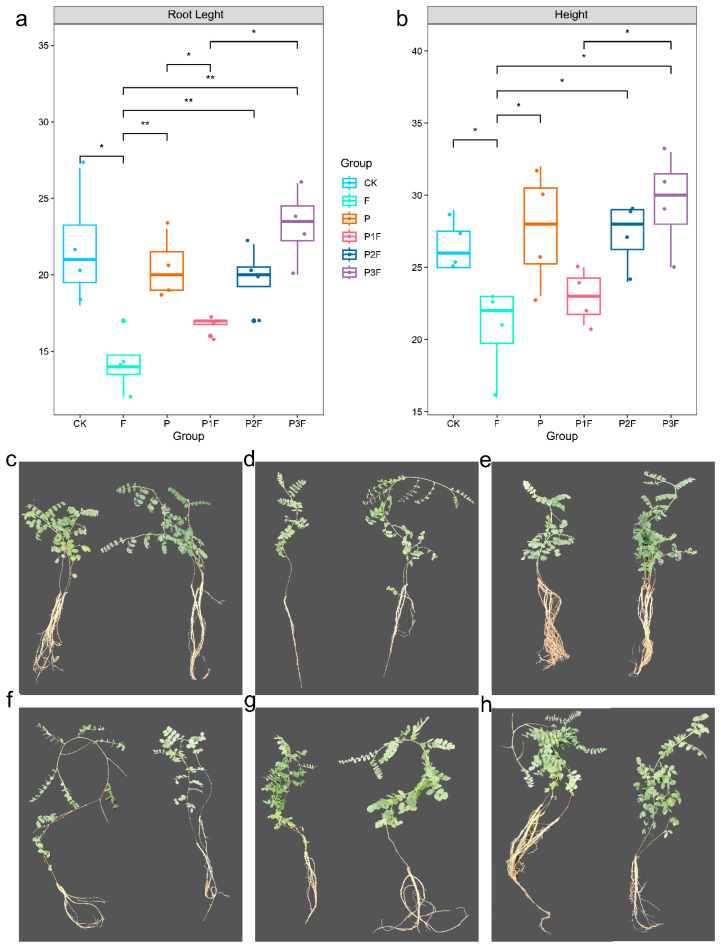
Root length (**a**) and stem height (**b**) of *A. membranaceus* seedlings under different treatments, as well as the growth status of seedlings under these treatments (**c**–**h**). The treatments include CK (blank control), F (*F. oxysporum* only), P (*P. halotolerans* only), and co-treatment with *F. oxysporum* and *P. halotolerans* at three spore concentrations: 1.0 × 10^5^ spores/mL (P1F), 5.0 × 10^5^ spores/mL (P2F), and 1.0 × 10^6^ spores/mL (P3F). Panels C–H show representative seedlings for each treatment: C (CK), D (F), E (P), and F–H (P1F, P2F, and P3F, respectively). Note: * indicates *p* < 0.05, ** indicates *p* < 0.01.

In order to further evaluate the antagonistic effect of *P. halotolerans* on *F. oxysporum*, this study adopted a pot experiment and set up six treatment groups, namely the blank control group (CK), the *F. oxysporum* alone treatment group (F), the *P. halotolerans* alone treatment group (P), and three co-inoculation treatment groups of *F. oxysporum* and different concentrations of *P. halotolerans* (P1F, P2F, P3F). The results showed that (Figure 5a–h), compared with the CK group, the F group significantly reduced the root length and plant height of *A. membranaceus*; and compared with the F group, the P group significantly increased the root length and plant height (Supplementary Table S5). In addition, compared with the F group, the higher concentrations of *P. halotolerans* (P2F and P3F treatments) also significantly increased the root length and plant height when co-inoculated with *F. oxysporum*. In conclusion, *F. oxysporum* significantly inhibited the growth of *A. membranaceus*, whereas *P. halotolerans* treatment partially alleviated pathogen-induced growth suppression.

### 3.6. Metabolic Differences in the Roots of A. membranaceus Associated with P. halotolerans Treatment

**Figure 6:**
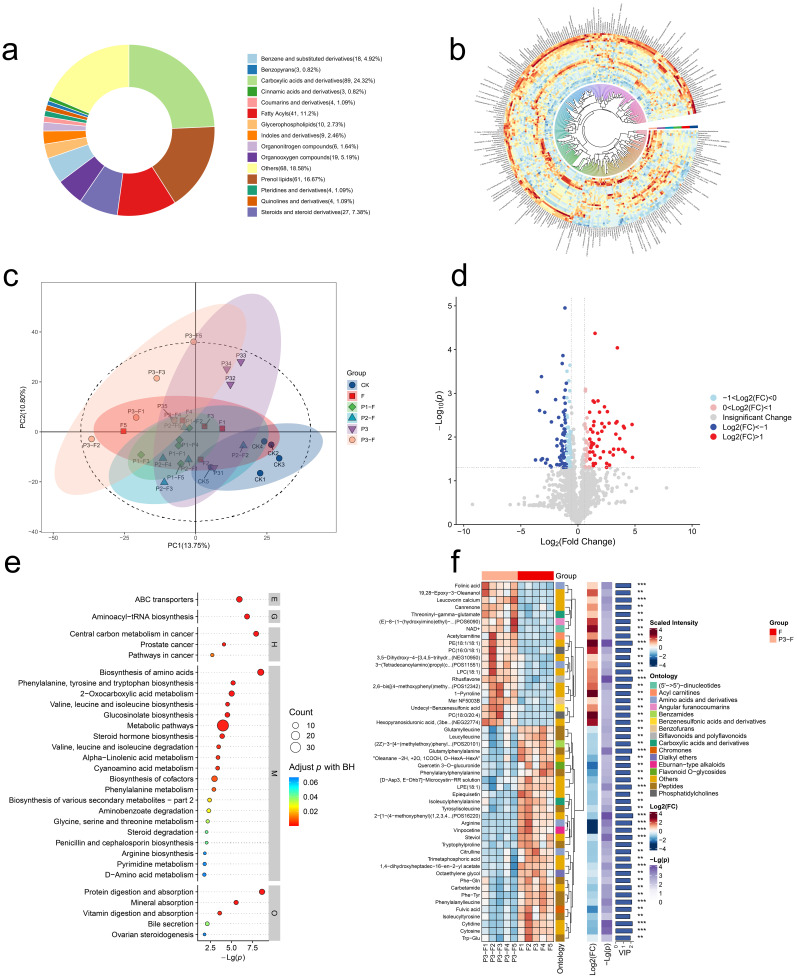
Comprehensive metabolite profiling and functional annotation of the root system of *A. membranaceus* seedlings under different treatments. (**a**) Circular diagram of HMDB class classification of root metabolites of A. membranaceus seedlings under 6 treatments (CK, F, P, P1F, P2F, and P3F); (**b**) Circular heatmaps of different metabolites processed differently; (**c**) PCA first and second principal components scatter plots for different treatments of all metabolites; (**d**) Volcanic map of differential metabolites between the F and P3F treatment groups. (**e**) Comparison of the KEGG pathway enrichment bubble plot of group F vs. P3F. (**f**) Comparative analysis of differential metabolite hierarchical clustering in the F. vs. CK treatment group, based on difference multiples (FC) and variable weight values (VIP) calculated using the OPLS-DA model. Note: Metabolism (M), Genetic Information Processing (G), Environmental Information Processing (E), Cellular Processes (C), Biological Systems (O), Human Diseases (H), Drug Development (D). Note: ** indicates *p* < 0.01, *** indicates *p* < 0.001.

To investigate the differences in metabolites of *A. membranaceus* after treatment with pathogenic fungi and antagonistic fungi, metabolomics analysis was performed. The results showed that all metabolites covered 14 categories, including Carboxylic acids and derivatives (24.32%), Prenol lipids (16.67%), Fatty Acids (11.20%), Steroids and steroid derivatives (7.38%), Organooxygen compounds (5.19%), Benzene and substituted derivatives (4.92%), Glycosphingolipids (2.73%), Indoles and derivatives (2.46%), Organonitrogen compounds (1.64%), Coumarins and derivatives (1.09%), Pteridines and derivatives (1.09%), Quinolines and derivatives (1.09%), Cinnamic acids and derivatives (0.82%), Benzopyrans (0.82%), and other metabolites (Figure 6a). The accumulation patterns and hierarchical clustering of metabolites were shown by a heat map, and it can be seen that there were the most differential metabolites between the F group and the P3F group (Figure 6b).

Principal component analysis (PCA) was performed on all metabolites (Figure 6c), indicating a clear separation of metabolites from different groups. Additionally, since the higher concentration of *P. halotolerans* (P3F group) showed the strongest suppressive effect under the experimental conditions, further analysis was conducted on the differential metabolites between the F group and P3F group, as shown in the volcano plot (Figure 6d). The results showed that a total of 227 differential metabolites were detected, of which 89 were upregulated and 138 were downregulated.

**Figure 7:**
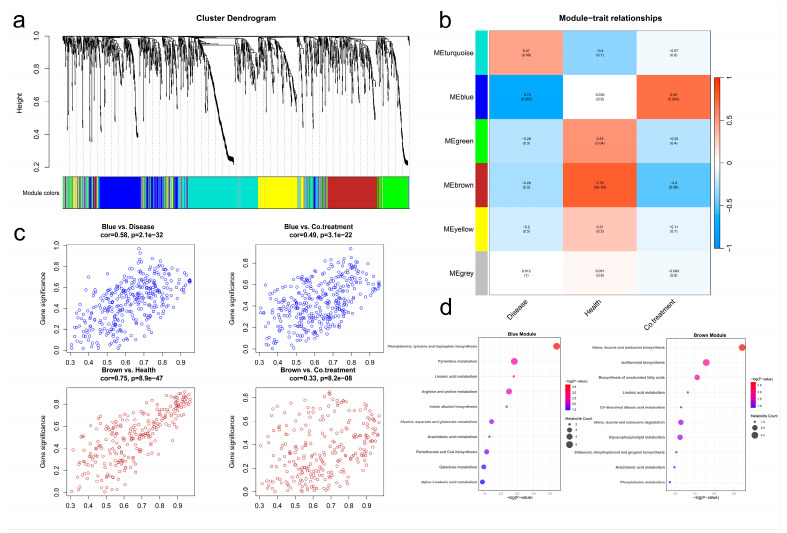
Metabolite module identification and KEGG pathway enrichment based on WGCNA. (**a**) Cluster dendrogram; (**b**) Heatmap of correlations between modules and treatment groups; (**c**) Scatter plots of metabolite significance in the blue and brown modules; (**d**) KEGG pathway enrichment analysis of metabolites in the blue and brown modules.

Further analysis was conducted on the differential metabolic pathways between the F group and the P3F group, and significant differences existed in multiple key metabolic pathways related to plant defense mechanisms between the two groups, such as ABC transporters, Aminoacyl-tRNA biosynthesis, Central Carbon Metabolism, Biosynthesis of amino acids, Protein digestion and absorption, and Mineral absorption (Figure 7a). The top 50 most important differential metabolites were screened by VIP value, and it was found that Arginine, Glutamylphenylalanine, LPE (18:1), Cytosine, Cytidine, Phenylalanylleucine, 1,4-dihydroxyheptadec-16-en-2-yl acetate, Steviol, Vinpocetine, and POS16220 in the P3F group were significantly down-regulated, while Rhusflavone, PE (18:1/18:1), Leucovorin calcium, and Folinic acid were significantly up-regulated (Figure 7b). These metabolic changes indicated clear differences in energy- and metabolism-related pathways between the F and P3F groups. In addition, several altered metabolites and pathways were associated with nutrient uptake- and defense-related metabolic processes.

### 3.7. WGCNA Revealed Distinct Metabolic Modules Associated with Different Treatments

To further characterize metabolite co-accumulation patterns among treatments, we conducted weighted gene co-expression network analysis (WGCNA) on 1524 detected metabolites (Supplementary Figure S2). The results showed that these metabolites were divided into six functional modules (Figure 7a). Among them, the blue module showed a significant negative correlation with the diseased group (cor = −0.58, *p* = 2.1 × 10^−32^), while it showed a significant positive correlation with the pathogen–antagonistic fungus co-treatment group (cor = 0.49, *p* = 3.1 × 10^−22^), indicating an association of this module with the co-treatment group (Figure 7b,c). On the other hand, the brown module showed a significant positive correlation with the healthy group (CK, cor = 0.75, *p* = 8.9 × 10^−47^), while the correlation with the pathogen–antagonistic fungus co-treatment group was significantly lower (cor = 0.33, *p* = 8.2 × 10^−8^), indicating that this module was more strongly associated with the healthy group (Figure 7b,c). Overall, distinct metabolic modules were associated with different treatment groups.

Further enrichment analysis was conducted on the metabolites in the blue and brown modules, and the results showed that the metabolites in the blue module were mainly enriched in pathways such as phenylalanine, tyrosine, tryptophan biosynthesis, and pyrimidine metabolism, suggesting that they may be related to nucleic acid metabolism and regulation of aromatic amino acid synthesis. The metabolites of the brown module were significantly enriched in the biosynthesis of valine, leucine, isoleucine, and isoflavone, indicating that they may play an important role in the metabolism of branched-chain amino acids and the synthesis of secondary metabolites (Figure 7d).

## 4. Discussion

### 4.1. The Occurrence of Root Rot Significantly Alters the Microbial Community Structure in the Rhizosphere Soil

An increasing amount of evidence indicates that the invasion of pathogenic fungi may alter the composition and community structure of soil microorganisms, thereby affecting the functions of the microbial community [36,37,38]. In the present study, root rot was associated with clear shifts in the rhizosphere microbial composition of *A. membranaceus*. The relative abundances of *Glomeromycota* and *Myxococcota* in the diseased soil decreased significantly (*p* < 0.05), while the relative abundances of *Basidiomycota* and *Fusarium*, the main pathogen causing root rot, increased significantly. It has been reported that most *Glomeromycota* are arbuscular mycorrhizal fungi, which can form arbuscular mycorrhizal symbioses with plant roots and depend on host plants for carbon and nutrient exchange [39]. Therefore, the reduction of *Glomeromycota* in diseased soil may be related to root damage and the consequent disruption of host–fungus interactions [40]. As decomposers, the survival and activities of Myxococcota may also be affected by the damage to plant roots or the reduction of root exudates [41]. The significant increase in the relative abundance of Basidiomycota may reflect changes in the rhizosphere environment following root damage, which could provide additional substrates for some decomposer-associated fungi [42,43]. In addition, some fungi reported in previous studies have been shown to inhibit root rot pathogens [44,45,46]. Taken together, these results suggest that root rot was associated not only with an increase in pathogen-related taxa, but also with a redistribution of fungal groups potentially involved in rhizosphere ecological balance and disease suppression. In this study, the occurrence of root rot significantly reduced the relative abundances of some beneficial microorganisms, such as *Mortierella*. It has been reported that *Mortierella* is associated with various positive effects on plant growth. It can improve the nutrient availability of the soil, especially the release and utilization efficiency of phosphorus, thereby helping plants to better absorb nutrients from the soil [47,48]. Accordingly, the decline of *Mortierella* in diseased soil may indicate a weakening of beneficial microbial functions related to nutrient acquisition and rhizosphere health.

Through the Mantel test of the fungal community and environmental factors, it was found that *Fusarium* had a strong correlation with ammonium nitrogen (NN) and available sulfur (AS). This result suggests that the distribution of *Fusarium* in the rhizosphere may be associated with variation in specific soil physicochemical factors. Studies by Maywald et al. have shown that when NN fertilizers are used, the incidence of root diseases in certain crops may increase, which is consistent with the strong correlation between *Fusarium* and NN [49]. Other studies have reported that when the soil contains a relatively high concentration of sulfur, it can promote plants to synthesize sulfur-rich defense compounds, such as glutathione and sulfur-containing amino acids, which may enhance plant defense responses and suppress the growth of pathogenic fungi [50]. Therefore, the correlation between *Fusarium* and AS observed here may reflect a potential link between sulfur availability, plant defense-related metabolism, and pathogen-associated rhizosphere variation. However, the present results are correlative and do not establish a direct causal relationship between individual soil factors and the abundance of *Fusarium*.

### 4.2. Root Rot Occurrence Was Associated with Increased Bacterial Network Complexity and Enhanced Positive Correlations in the Fungal Community Network

In this study, we observed that the occurrence of root rot disease was associated with changes in the co-occurrence network pattern, leading to an increase in the average degree and number of edges of network nodes. This suggests that root rot occurrence was associated with the reorganization of microbial communities. Wang et al. found that in the rhizosphere soil of plants infected with cucumber root rot, the complexity of microbial networks, the average degree of nodes, and the number of edges significantly increased [51]. This phenomenon may be associated with the proliferation of pathogens and concurrent shifts in rhizosphere microorganisms, which could alter community composition and network structure [52]. In addition, modularity has been regarded as an important topological property of ecological networks and has often been discussed in relation to network stability [53], reflecting the independent interactions and functional differentiation of different species communities in ecosystems [54,55]. This phenomenon is particularly evident in bacterial community networks, where the modularity index of diseased bacterial community networks is significantly lower than that of healthy networks. In the present study, this pattern may indicate that disease was associated with a weakening of bacterial network compartmentalization and a reorganization of specific microbial interaction modules under pathogen pressure [56].

In fungal community networks, the positive interactions in healthy networks are significantly lower than those in diseased networks. This observation aligns with Wang et al., who found that root rot infection significantly increases the positive interactions within the *Panax notoginseng* fungal community network [56]. Faust and Raes suggested that positively connected communities in microbial networks may be less stable, whereas negative correlations are often discussed as being associated with competitive relationships that can contribute to community balance [57]. These positive correlations may reflect altered fungal network organization under disease pressure, which could be associated with pathogen proliferation [58]. In bacterial networks, the opposite situation is observed, where the occurrence of root rot promotes the growth of negative interrelationships in the network. This pattern may reflect a shift in bacterial interaction structure under disease pressure, rather than a direct indication of specific ecological functions. Increased negative correlations in bacterial networks may reflect changes in bacterial interaction patterns under disease pressure, such as competition and resistance expression [59]. Accordingly, the contrasting trends observed in fungal and bacterial networks may indicate different modes of microbial network reorganization in response to root rot. This change may represent an attempt by plant microbial communities to maintain a certain functional balance, even if it is in a non-ideal state under disease pressure [36,60,61]. Overall, root rot occurrence was associated with enhanced positive correlations in the fungal community network and increased negative correlations in the bacterial community network, suggesting distinct patterns of microbial network reorganization under disease pressure.

### 4.3. Metabolomic Insights into Host Responses Associated with P. halotolerans Treatment in A. membranaceus Under F. oxysporum Challenge

In this study, we screened a fungal strain showing strong antagonistic activity against the causal agent of Astragalus root rot, *F. oxysporum*, and identified it as *P. halotolerans*. In further pot experiments, it was shown that *P. halotolerans* exerted a suppressive effect on root rot symptoms under the experimental conditions. These results supported the selection of *P. halotolerans* for further metabolomic analysis of host responses under pathogen challenge. To explore host metabolic responses associated with *P. halotolerans* treatment, we conducted plant metabolomics analysis. Compared with the F treatment group (the pathogenic fungus treatment group), the P3F treatment group (the pathogen antagonistic fungi co-treatment group) showed significant differences in multiple key metabolic pathways related to plant response processes, including ABC transporters, aminoacyl-tRNA biosynthesis, central carbon metabolism, biosynthesis of amino acids, protein digestion and absorption, and mineral absorption. The top 50 differential metabolites between the F group and the P3F group were screened based on VIP values, and these differential metabolites were analyzed. Among these metabolites, Arginine, Glutamylphenylalanine, LPE(18:1), Cytosine, Cytidine, Phenylalanylleucine, 1,4-dihydroxyheptadec-16-en-2-yl acetate, Steviol, and Vinpocetine were significantly downregulated in the P3F group, while Rhusflavone, PE (18:1/18:1), Leucovorin calcium, and Folinic acid were significantly upregulated. Arginine is a key amino acid in nitrogen metabolism, while Cytosine and Cytidine are important substances in the nucleic acid synthesis pathway related to plant pathogen resistance [61,62]. Their altered abundance in the P3F group may reflect shifts in host metabolic allocation under pathogen challenge and antagonist treatment [63]. LPE (18:1) and PE (18:1/18:1) are both important components of the cell membrane [64,65]. The downregulation of LPE and upregulation of PE may indicate changes in membrane lipid metabolism associated with host responses to *P. halotolerans* treatment. The increase in PE may be associated with the maintenance of membrane integrity and functionality [66,67]. Research has found that folate metabolism plays a crucial role in plant disease resistance and DNA repair. Enhancing folate metabolism can improve both disease resistance and genomic stability in plants [68,69]. In this study, the significant upregulation of leucovorin calcium and folinic acid may suggest the involvement of folate-related metabolic pathways in the host response to pathogen challenge [70,71].

Through WGCNA, we further identified functional modules associated with root rot disease. This analysis provided an additional systems-level view of the metabolite changes associated with pathogen infection and *P. halotolerans* treatment. Among them, the blue module showed a significant negative correlation with the diseased group, while exhibiting a positive correlation with the pathogen-antagonist co-treatment group. Enrichment analysis revealed that the metabolites in this module were mainly enriched in metabolic pathways such as phenylalanine, tyrosine, and tryptophan biosynthesis, as well as pyrimidine metabolism. This suggests that root rot was associated with marked changes in nucleic acid metabolism and the biosynthesis of aromatic amino acids [72,73]. Aromatic amino acids not only serve as fundamental building blocks for proteins but also act as precursors for the biosynthesis of various secondary metabolites, playing a pivotal role in plant growth, development, and stress adaptation [74]. The positive association of this module with the co-treatment group suggests that *P. halotolerans* treatment was accompanied by shifts in pathogen-associated metabolic patterns, particularly those related to nucleic acid metabolism and aromatic amino acid biosynthesis. The brown module exhibited a significant positive correlation with the healthy group but showed relatively low correlation with the pathogen-antagonist co-treatment group. Enrichment analysis revealed that metabolites in this module were predominantly enriched in biosynthetic pathways of valine, leucine, isoleucine, and isoflavonoids, suggesting their possible role in maintaining the healthy state of *A. membranaceus* [75,76]. By contrast, the weaker association of this module with the co-treatment group suggests that antagonist-treated plants did not fully converge with the metabolic profile of the healthy group. Notably, the metabolic profile of antagonist-treated plants differed from that of the healthy group, indicating that while *P. halotolerans* showed a suppressive effect on root rot symptoms, the metabolic patterns it induced were not identical to those of the natural healthy state.

This study reveals that root rot caused by *F. oxysporum* significantly disrupts the rhizosphere microbial community and plant metabolism in *A. membranaceus*, leading to reduced microbial diversity and destabilized microbial networks. A fungal strain with antagonistic activity, *P. halotolerans*, was identified from diseased soils and showed inhibitory activity against *F. oxysporum* in vitro as well as a suppressive effect in pot experiments. Metabolomic analysis suggested that *P. halotolerans* treatment was associated with changes in host pathways related to energy metabolism, membrane lipid metabolism, and nutrient uptake, while also being associated with altered aromatic amino acid and pyrimidine metabolism under pathogen challenge. These findings provide candidate microbial resources and initial evidence that the suppressive effect of *P. halotolerans* may be accompanied by host metabolic reprogramming associated with rhizosphere disease suppression in *A. membranaceus*. However, further studies are still needed to evaluate biosafety, metabolite safety, and the feasibility of practical application.

## 5. Conclusions

This study showed that root rot caused by *F. oxysporum* was associated with marked changes in the rhizosphere microbial community and plant metabolism of *A. membranaceus*, including reduced microbial diversity and altered microbial network structure. A fungal strain with antagonistic activity, *P. halotolerans*, was isolated and identified, and it showed inhibitory activity against *F. oxysporum* in vitro as well as a suppressive effect on root rot symptoms in pot experiments under the experimental conditions. Metabolomic analysis suggested that *P. halotolerans* treatment was associated with changes in host pathways related to energy metabolism, membrane lipid metabolism, nutrient uptake, and other defense-associated processes, while also being associated with altered aromatic amino acid and pyrimidine metabolism under pathogen challenge. Together, these findings provide candidate microbial resources and preliminary mechanistic insights into rhizosphere-associated suppression of Astragalus root rot. Further studies are still needed to evaluate the biosafety, metabolic safety, and practical application feasibility of *P. halotolerans*.

## Data Availability

The raw sequencing data have been deposited in the SRA (BioProject PRJNA1310244).

## Supplementary Materials

The following supporting information can be downloaded at: https://www.mdpi.com/article/10.3390/jof12040283/s1, Figure S1: Topological roles of fungal ASVs in the co-occurrence network based on among-module connectivity (Pi) and within-module connectivity (Zi); Figure S2: Analysis of network topology for soft-threshold power selection in WGCNA; Table S1: Topological properties of bacterial and fungal co-occurrence networks under diseased and healthy conditions; Table S2: Pairwise associations among ASVs with correlation type, strength, and significance in the microbial co-occurrence network; Table S3: Molecular identification of microbial isolates based on 16S rRNA and ITS region sequence similarity; Table S4: Antagonistic effect of candidate strains against pathogen BN2-1; Table S5: Root length (A) and stem height (B) of *A. membranaceus* seedlings under different treatments.
