# Supplementary figures and images for: Characterization of Penicillium halotolerans with Antagonistic Activity Against Fusarium Root Rot in Astragalus membranaceus
Source: J Fungi (Basel). 2026 Apr 17;12(4):283. doi: 10.3390/jof12040283 (PMC13117717; doi:10.3390/jof12040283)

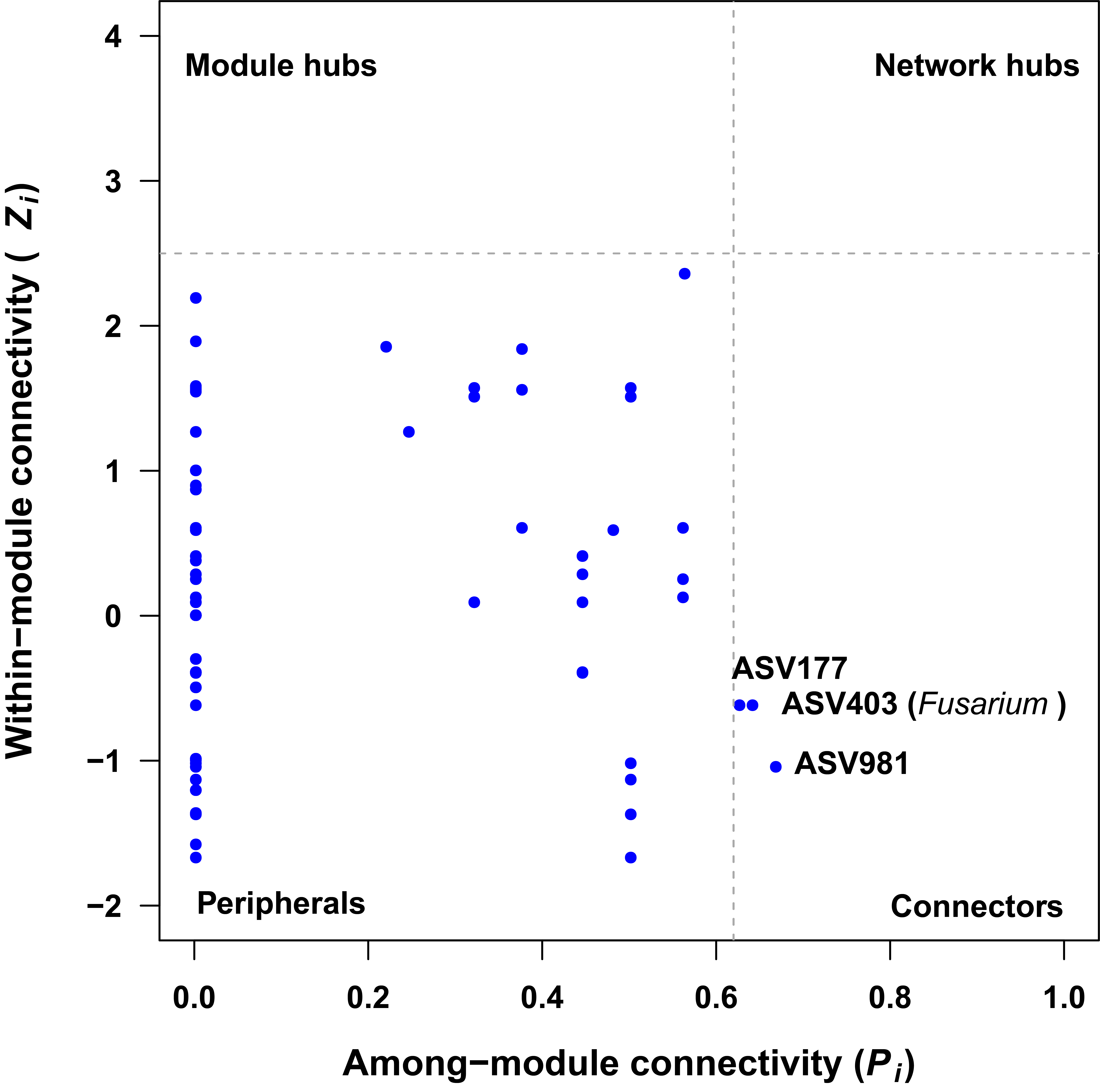

Supplement: Supplementary file 1 [file jof-12-00283-s001.zip › Supplementary Figure S1.png]

### Scale independence

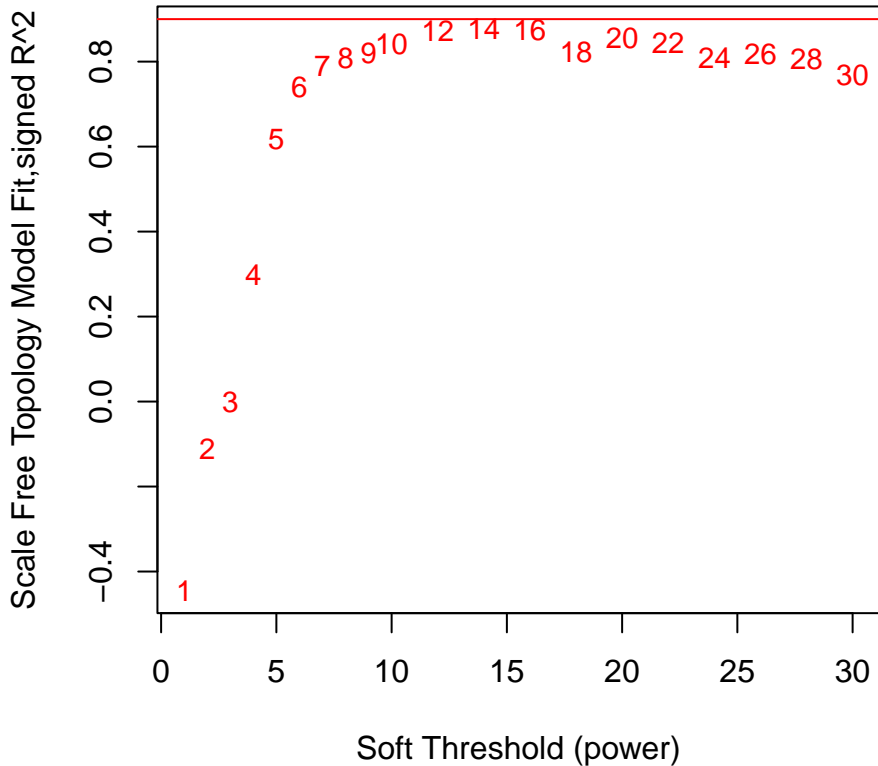

### Mean connectivity

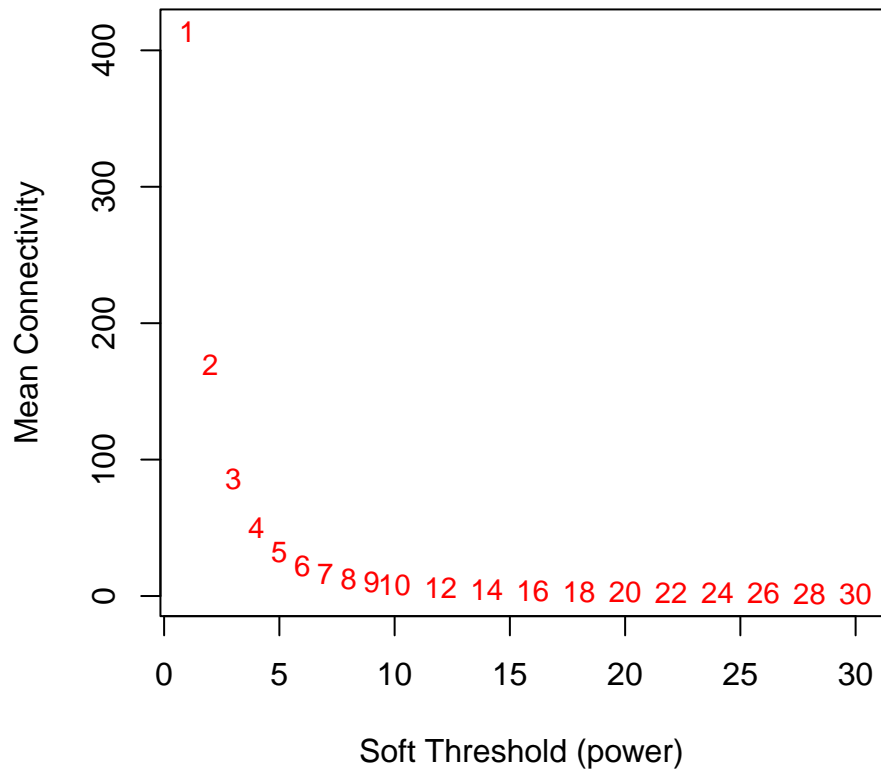

Supplement: Supplementary file 1 [file jof-12-00283-s001.zip › Supplementary Figure S2.pdf]
